# Auxeticity Tuning by Nanolayer Inclusion Ordering in Hard Sphere Crystals

**DOI:** 10.3390/ma17184564

**Published:** 2024-09-17

**Authors:** Jakub W. Narojczyk, Krzysztof W. Wojciechowski, Jerzy Smardzewski, Konstantin V. Tretiakov

**Affiliations:** 1 Institute of Molecular Physics, Polish Academy of Sciences, M. Smoluchowskiego 17, 60–179 Poznań, Poland; kww@ifmpan.poznan.pl; 2 Uniwersytet Kaliski im. Prezydenta St. Wojciechowskiego, Nowy Świat 4, 62-800 Kalisz, Poland; 3 Department of Furniture Design, Faculty of Forestry and Wood Technology, Poznan University of Life Sciences, Wojska Polskiego 28, 60-637 Poznań, Poland; jsmardzewski@up.poznan.pl

**Keywords:** auxetics, negative Poisson’s ratio, nanolayers, hard spheres inclusions, Monte Carlo simulations

## Abstract

Designing a particular change in a system structure to achieve the desired elastic properties of materials for a given task is challenging. Recent studies of purely geometrical atomic models have shown that structural modifications on a molecular level can lead to interesting and desirable elastic properties. Still, the result of such changes is usually difficult to predict. The present work concerns the impact of nanolayer inclusion ordering in hard sphere crystals on their elastic properties, with special attention devoted to their auxetic properties. Two sets of representative models, based on cubic crystals consisting of 6×6×6 unit cells of hard spheres and containing either neighboring or separated layers of spheres of another diameter, oriented orthogonally to the [001] direction, have been studied by Monte Carlo simulations in the isothermal–isobaric (*NpT*) ensemble. Their elastic constants have been evaluated using the Parinello–Rahman approach. The Monte Carlo simulations showed that introducing the layer inclusions into a pure face-centered cubic (FCC) structure leads to the system’s symmetry changes from cubic symmetry to tetragonal in both cases. Essential changes in the elastic properties of the systems due to layer ordering were found both for neighboring and separated inclusions. It has been found that the choice of a set of layer inclusions allows one to tune the auxetic properties in two crystallographic directions ($\left[110\right]\left[1\bar{1}0\right]$ and $\left[101\right]\left[\bar{1}01\right]$). In particular, this study revealed that the change in layer ordering (from six separated layers to six neighboring ones) allows for, respectively: (i) enhancing auxeticity of the system in the $\left[101\right]\left[\bar{1}01\right]$ direction with almost loss of auxetic properties in the $\left[110\right]\left[1\bar{1}0\right]$ direction in the case of six separated layers, while (ii) in the case of six neighboring layers, keeping the auxetic properties in both auxetic directions independently of the size of spheres constituting inclusions.

## 1. Introduction

Poisson’s ratio [1] (PR, ν) is one of the oldest and most commonly used parameters describing a material’s behavior under loading. Based on its value for a given material, one can know how much the latter will shrink or widen its transverse dimensions while stretched longitudinally. The theory of elasticity [1] predicts the boundaries for the PR for 3D isotropic materials in the 1/2 to −1 region. Despite the theoretical prediction, the “negative shrinkage” of the body’s transverse dimensions was thought to be impossible for a long time. It was not until the late 1980s when the first manufactured material with a negative Poisson’s ratio was fabricated [2] and the first thermodynamic model showing such a phenomenon was formulated and solved [3,4,5]. Thus, *auxetics* [6], as they came to be called, are a relatively new class of materials with extraordinary and counter-intuitive elastic properties. Over the last four decades, an ever-growing interest in auxetics has been observed among scientists and engineers. Several model materials with auxetic properties have been proposed [7,8,9,10,11,12,13,14,15,16,17,18,19]. Materials exhibiting a negative Poisson’s ratio have been fabricated (among others) in the form of polymers [20,21], composites [22,23], foams [24,25,26], fabrics [27,28,29,30], and metamaterials [31]. Auxetic properties were observed in existing materials, e.g., in about 69% of metals with a cubic crystallographic structure [32]. Despite the intense studies of materials with negative PR, a deep understanding of the origin of these fascinating elastic properties is still required for a wide practical application of auxetics [33,34]. Thus, studying various physical properties of simple models of materials can provide us with the necessary information and deeper insight into the physical phenomena required for designing new materials with extraordinary properties or modifying the given properties of existing materials. For example, such simple atomic models are very helpful in explaining the origin of auxeticity in cubic metals [32] and basic models in condensed matter physics like hard and soft sphere crystals [35,36]. Beyond that, simple models also allow us to investigate the impact of structure modification on elastic properties at the micro level [35,36,37].

Designing new materials with the required elastic properties is tedious and costly. Structural modifications of existing materials to tailor their properties for specific applications may be more efficient. However, to make this possible, a broader knowledge of how such modifications on the micro level impact macroscopic properties is required. Thus, the study of simple atomic systems like hard sphere crystals plays an essential role in searching for mechanisms behind, e.g., their auxetic properties. The model of hard sphere crystals is one of the simplest and most fundamental ones—it is widely used in the theory of liquids [38] and condensed matter physics [39]. A number of works [35,40,41,42,43,44,45,46] have been devoted to the elastic properties of hard sphere crystals. Most importantly, the FCC hard sphere crystal is a partially auxetic system [47]. Its Poisson’s ratio in the $\left[110\right]\left[1\bar{1}0\right]$ direction is −0.054(9) at the melting density [41] and −0.072(1) in the close packing limit [36].

Studies of model auxetics consisting of molecules interacting through hard potential were initiated by Wojciechowski [3,4,48] and coworkers [5,49,50,51,52,53] and are currently continued by Tretiakov’s group. Recently, a pioneering approach for designing materials at the atomic level by modifying their microcrystalline structure was proposed [37,54,55,56,57,58]. In this approach, the elastic properties of the model systems are evaluated using Monte Carlo simulations. Studies carried out to date that utilized nanochannel inclusions have shown that one can strongly enhance [56] or eliminate [59] auxetic properties when introducing single or triple nanochannel inclusions, respectively. Moreover, recent studies showed that proper choice of the number and the orientation of nanochannel inclusions can enhance crystal stiffness [58]. Nevertheless, despite the number of studies completed so far, the influence of microscopic modifications on macroscopic elastic properties is difficult to predict. It has been shown that one can eliminate auxetic properties from the hard sphere crystal [60] by introducing a joined [001] nanochannel and (001) nanolayer inclusion. This is an interesting finding because, when used separately, these inclusions enhance auxetic properties, either strongly [56] or slightly [61]. The latter investigation (that revealed only a slight enhancement to auxetic properties) left open some important questions regarding the influence of layer inclusions on elastic properties. Thus, in this work, we focus our attention on hard sphere crystals containing multiple planar hard sphere inclusions oriented parallel to each other and orthogonal to the [001] direction, which has not been studied before. *Remark:* It is worth adding that very simple auxetic models with hard interactions with *one* degree of freedom have been studied by various researchers. It is also worth mentioning the works by Grima and coworkers on identical rigid rotating units [14,62,63,64,65,66,67,68], as well as, the works of Lim who proposed rigid models composed of two or more rigid rotating units [69,70,71].

This work aims to study the elastic properties of hard sphere crystals with multiple nanolayers oriented orthogonally to the [001] direction and formed by hard spheres of another diameter. Particularly, the influence of an increasing number of inclusion layers on the elastic properties of the system is investigated. Another important aspect is the impact of layer inclusion ordering on the elastic (overall) and the auxetic (in particular) properties of the system. Studies of such models can be interesting from a practical point of view. As a consequence of the rapid development in nanotechnology, one can soon expect the manufacturing of metamaterials based on the ideas presented in this work using, e.g., atomic layer deposition [72].

The structure of this article is as follows. In Section 2, the studied models are described. The research method is briefly reviewed in Section 3. The discussion of the results is found in Section 4, whereas Section 5 contains the summary and conclusions.

## 2. The Model

The model system consists of *N* spheres of the diameter σ, initially forming an FCC crystal. The spheres interact with their nearest neighbors with a purely geometrical hard sphere (HS) potential of the form:

(1) $$\beta u_{ij}=\left\{\begin{array}{cc} \infty , & r_{ij}<\sigma_{ij},\\ 0, & r_{ij}\geq \sigma_{ij}, \end{array}\right.$$

where $r_{ij}$ is the distance between centers of spheres *i* and *j*; $\sigma_{ij}$ stands for the sum of the radii of these spheres; $\beta =1/(k_{B}T)$; $k_{B}$ is the Boltzmann constant; and *T* is the temperature. Into this crystal, we introduce an inclusion by replacing all spheres in selected crystallographic planes orthogonal to the [001] direction with other hard spheres. The replaced spheres differ from the original spheres only in size. The inclusion spheres have a diameter equal to $\sigma^{\prime }$, such that $\sigma^{\prime }\neq \sigma$. To analyze the impact of inclusions on the elastic properties of the studied models, we introduce a parameter $\sigma^{\prime }/\sigma$, which measures the ratio of diameters of the inclusion and matrix spheres.

The proposed model was considered in periodic boundary conditions. Thus, one obtains a system with a periodic stack of inclusion nanolayers that are infinite in the *x*–*y* plane. The *N* particles within the periodic box are thought of as the unit supercell. It is important to note that, in each case when $\sigma^{\prime }\neq \sigma$, the resulting system is *no longer cubic*. The case when only one inclusion layer is present in the unit supercell was studied in [61]. It is important to note that, in the present article, as was done in [61], the simulations are restricted to representative supercells corresponding to 6×6×6 unit cells of the FCC structure. Here, however, we consider the systems with multiple nanolayer inclusions per unit supercell.

**Figure 1:**
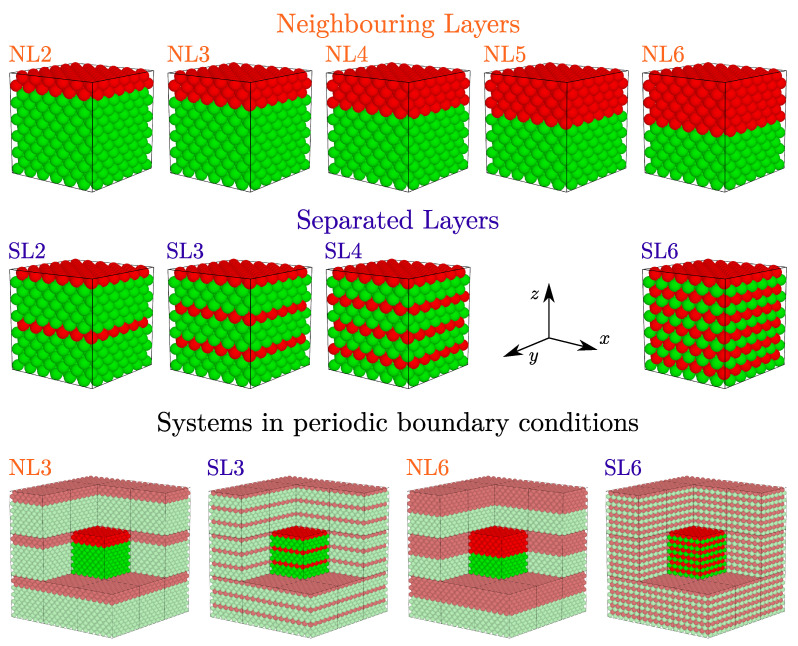
The geometry of studied systems containing from one to six nanoinclusion layers in various configurations. The top in the figure represents the systems with neighboring layer inclusions. The middle part of the figure represents the systems with separated layer inclusions. The red spheres represent the inclusion ones, whereas the green spheres represent the ‘matrix’ ones. At the bottom of the figure, four selected systems in the periodic boundary conditions are presented, where supercell (bright colors) and periodic images of the supercell (pale colors) are shown. Some of the periodic images, in the line of sight, have been removed to facilitate the presentation.

The total number of inclusion spheres within the supercell is $N_{\mathrm{inc}}=2N_{x}\cdot N_{y}\cdot L$, where $N_{x}$ and $N_{y}$ correspond to the number of FCC cells in respective directions, and where *L* is the number of single inclusion layers introduced into the system. The ratio $N_{\mathrm{inc}}/N$ will be referred to as the concentration of the inclusion particles and denoted by *c*. We studied systems with *L* between 2 and 6 to investigate the influence of the increasing concentration of inclusion particles on the elastic properties of the system as well as the impact of the layer inclusion ordering on the auxetic properties of the system at the same concentration. The spatial localization of *L* layers within the unit supercell could be arbitrary, but with the increase in *L*, the number of possible structures increases rapidly. So, we have chosen to study two cases: (i) when the inclusion layers are located next to each other, and (ii) when the individual inclusion layers are separated by at least one layer of original spheres with diameter σ. The first case is further referred to as Neighboring Layers (NLs) and the second one as Separated Layers (SLs). The studied SL systems are not the only possible cases that can be obtained for a given *L*. The reason for this choice is that these are the only ones for which the ordering is periodic within the simulation box and, additionally, systems with the same concentration (*c*) can be compared. Visualization of the studied systems is presented in Figure 1, and their details are gathered in Table 1, where graphical inserts indicate the particular layer inclusion ordering within the unit supercell.

## 3. The Method

### 3.1. Elastic Properties

Monte Carlo (MC) computer simulations in the isobaric–isothermal ensemble (NpT) were performed. The Parinello–Rahman method [49,73,74] was used to determine the elastic properties of the studied systems. According to this method [74], the Lagrange strain tensor can be expressed by the box matrix h and the reference box matrix $h_{p}$ in the following form:

(2) $$\varepsilon =\frac{1}{2}\left(h_{p}^{-1}.h.h.h_{p}^{-1}-I\right)\;,$$

where I is a unit matrix. The matrix h is a symmetric matrix formed by the edge vectors of the simulation box. The reference matrix, defined as $h_{p}\equiv \langle h\rangle$, is the average value of the h matrix over the NpT ensemble at equilibrium under dimensionless pressure $p^{*}=p\beta \sigma^{3}$. The knowledge of the Lagrange strain tensor fluctuations allows one to calculate all components of the elastic compliance tensor using the formula [49]:

(3) $$S_{\alpha \beta \gamma \delta }=\beta V_{p}\langle \Delta \varepsilon_{\alpha \beta }\Delta \varepsilon_{\gamma \delta }\rangle \;,$$

where $V_{p}=\left|\det\left(h_{p}\right)\right|$ is the volume of the system at pressure *p* at equilibrium, $\Delta \varepsilon_{\alpha \beta }=\varepsilon_{\alpha \beta }-\langle \varepsilon_{\alpha \beta }\rangle$, $\langle \varepsilon_{\alpha \beta }\rangle$ is the average over the NpT ensemble, and α,β,γ,δ = *x*, *y*, or *z*.

Poisson’s ratio is an arbitrary direction and, for any given symmetry, calculated directly from the elastic compliance tensor by the following expression [75]:

(4) $$\nu_{nm}=-\frac{m_{\alpha }m_{\beta }S_{\alpha \beta \gamma \delta }n_{\gamma }n_{\delta }}{n_{\zeta }n_{\eta }S_{\zeta \eta \kappa \lambda }n_{\kappa }n_{\lambda }}\;.$$

From the expression above, it follows that Poisson’s ratio depends on the choice of two mutually orthogonal directions (represented as unit vectors): $\vec{n}$, which indicates the direction of the applied external stress, and $\vec{m}$, which corresponds to the direction in which PR is measured. According to the definition of Poisson’s ratio, $\vec{m}$ is always orthogonal to $\vec{n}$. Here, it should be noted that the Einstein summation convention is used on Greek indices.

As a final remark, in this work, in order to describe the elastic properties of studied systems, we used the elastic constants tensor ($B_{\alpha \beta \gamma \delta }$) calculated from the $S_{\alpha \beta \gamma \delta }$ tensor as follows [76]:

(5) $$\sum_{n,m}S_{ijmn}B_{mnkl}=\frac{1}{2}\left(\delta_{ik}\delta_{jl}+\delta_{il}\delta_{jk}\right),\;$$

where $\delta_{ij}$ is the Kronecker delta. Further, we expressed the elastic constants tensor with the *elastic constants matrix* $B_{ij}$ using the Voigt representation [77]. The Latin indices for the $B_{ij}$ elements of this symmetric square matrix take the values i,j=1,...,6.

### 3.2. Computation Details

In this study, the systems of N=864 particles have been investigated. It has been shown recently that, for this size of system and for that kind of calculation, the dependence of the obtained results on the size of the studied system is negligible [58]. Therefore, all further presented results refer to systems with inclusions, consisting of 864 spheres. This corresponds to the reference FCC crystal of 6×6×6 unit cells. In such a system, a single inclusion layer consists of 72 spheres. Thus, we have considered systems with the concentration of inclusion particles *c* in the range between 8.13% and 50% (see Table 1). The systems were investigated under constant pressure $p^{*}=50$ as the representative one. The impact of the inclusion sphere diameter on elastic properties has been investigated in the range of $\sigma^{\prime }/\sigma$ between 0.95 and 1.06, for which the tetragonal system remained stable at considered pressure. Earlier, it has been shown that single nanolayer inclusions introduced into the FCC hard sphere crystal (oriented orthogonally to the [001] direction) lower the symmetry from cubic to tetragonal [61]. The results presented in the present work show only the cases for given parameters ($p^{*}$, $\sigma^{\prime }/\sigma$) when the stable system with tetragonal symmetry was observed. The elastic constants of each system and Poisson’s ratio were obtained as an average from at least 100 independent simulations under each thermodynamic condition. Every simulation that was run lasted for $10^{7}$ MC cycles. The first 10% of the latter in every simulation was treated as the period in which the system reached thermodynamic equilibrium and was discarded from calculations of elastic constants.

## 4. Results and Discussion

**Figure 2:**
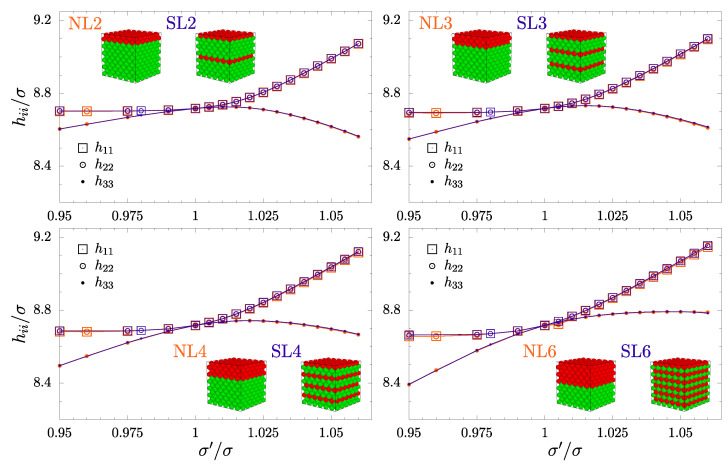
The comparison of diagonal periodic box matrix elements $h_{ii}$ for systems with the same number of neighboring (NL) and separated (SL) nanolayers. The data are plotted against $\sigma^{\prime }/\sigma$, which is the ratio of diameters of the inclusion and the matrix spheres. The orange symbols, representing NLs, are slightly larger than the blue symbols, which represent SLs.

To achieve the goal of this work, it is necessary to analyze the impact of adding consecutive layer inclusions (from L=2 to 6) on the elastic properties of hard sphere crystals. Due to hard potential, all the system particles interact purely geometrically. Thus, only when the $\sigma^{\prime }/\sigma$ value changes can any impact of the inclusions be observed. In Figure 2, the diagonal components of the h matrix are presented. The results are plotted for pairs of systems (SL and NL) with a given number of inclusion layers and show that the size and shape of the sample are not affected by the spatial ordering of the inclusion layers. The shape of the simulation box when $\sigma^{\prime }/\sigma \neq 1$ is a parallelogram. The off-diagonal elements of the box matrix fluctuate around zero, and their averages are many orders of magnitude less than the diagonal elements and thus treated as zero, similarly to previous work [61]. It can be seen in Figure 2 that, with the increase in $\sigma^{\prime }/\sigma$, the increasing in-plane dimensions of inclusions are reflected in the increase in $h_{11}$ and $h_{22}$. For systems with a small number of inclusion layers (from 2 to 4), the horizontal expansion of the supercell (in the plane of the inclusion) is accompanied by a decrease in $h_{33}$ (the direction orthogonal to the inclusion plane) at larger values of $\sigma^{\prime }/\sigma$, compared to the case when $\sigma^{\prime }/\sigma =1$. For systems with six nanolayers, this effect is balanced with the increase in the thickness of the combined inclusion and thus, we observe a small increase in $h_{33}$ with the increasing value of $\sigma^{\prime }/\sigma$. On the other hand, the decrease in $h_{33}$ when the diameters of inclusion spheres become smaller than those of the matrix spheres ($\sigma^{\prime }/\sigma <1$) is (as expected) directly proportional to the number of inclusion layers (*L*).

**Figure 3:**
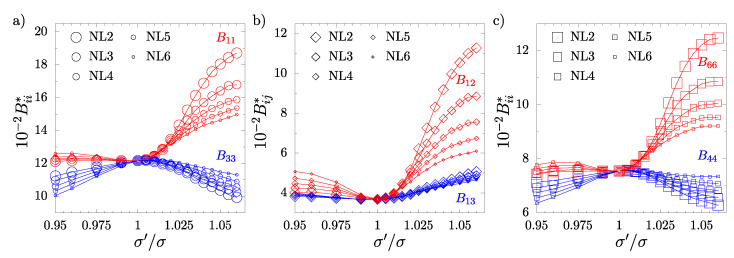
The elastic constants ($B_{ij}^{*}$) of systems with neighboring nanolayers (NLs) as a function of $\sigma^{\prime }/\sigma$: (**a**) $B_{11}^{*}$ and $B_{33}^{*}$, (**b**) $B_{12}^{*}$ and $B_{13}^{*}$, (**c**) $B_{44}^{*}$ and $B_{66}^{*}$.

**Figure 4:**
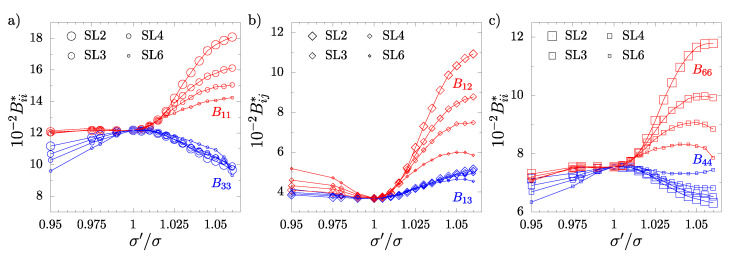
The elastic constants ($B_{ij}^{*}$) of systems with separated nanolayers (SLs) as a function of $\sigma^{\prime }/\sigma$: (**a**) $B_{11}^{*}$ and $B_{33}^{*}$, (**b**) $B_{12}^{*}$ and $B_{13}^{*}$, (**c**) $B_{44}^{*}$ and $B_{66}^{*}$.

It should be noted that, in all cases when $\sigma^{\prime }/\sigma \neq 1$, the systems with inclusions do not exhibit cubic symmetry. This is also seen in Figure 3 and Figure 4 for NL and SL systems, respectively, where the non-zero elastic constants $B_{ij}$ as a function of $\sigma^{\prime }/\sigma$ have been plotted. The analysis of the elastic constants matrices (B) for all the considered systems with $\sigma^{\prime }/\sigma \neq 1$ has shown that all those systems have tetragonal symmetry (422 symmetry class [77]). For this symmetry, there are six independent elastic constants. The analysis of the obtained results confirms that the following relations required for tetragonal symmetry [77] are fulfilled: $B_{11}=B_{22}$, $B_{44}=B_{55}$, $B_{13}=B_{23}$, and $B_{ij}=0$ for i=1,...,5, j=4,5,6, i≠j, similar to a previous study [61]. Thus, the elastic constant matrix has the following form:

(6) $$B=\left[\begin{array}{cccccc} B_{11} & B_{12} & B_{13} & 0 & 0 & 0\\ \cdot & B_{11} & B_{13} & 0 & 0 & 0\\ \cdot & \cdot & B_{33} & 0 & 0 & 0\\ \cdot & \cdot & \cdot & B_{44} & 0 & 0\\ \cdot & \cdot & \cdot & \cdot & B_{44} & 0\\ \cdot & \cdot & \cdot & \cdot & \cdot & B_{66} \end{array}\right]\;.$$

In Figure 3 and Figure 4, it is also seen that both the number of inclusion layers and their ordering exerts a significant impact on the elastic properties of the hard sphere crystal. It can be noted that: (i) the most significant changes with respect to the system without inclusions ($\sigma^{\prime }/\sigma =1$) are observed for systems with the smallest number of included nanolayers, and (ii) the $B_{11}$, $B_{12}$, and $B_{66}$ constants increase more in the neighboring nanolayer (NL) systems than in the SL systems. In general, the more nanolayers are introduced into the unit supercell, the weaker the impact of inclusions on the elastic constants. One can observe some tendency of the values of the elastic constants of both NL and SL systems to those of the cubic system with increases in *L*. In particular, $B_{44}$ for NL6 (Figure 3c) and SL6 (Figure 4c) systems are very close to the cubic case when $\sigma^{\prime }/\sigma >1$. Interestingly, the opposite behavior is observed when $\sigma^{\prime }/\sigma <1$. The elastic constants decrease with the number of nanolayers introduced into the system. The $B_{13}$ constants in both NL and SL systems show weak changes in their values for $\sigma^{\prime }/\sigma <1$ and a slight increase for $\sigma^{\prime }/\sigma >1$. Moreover, they show faint dependence on the number of layer inclusions.

**Figure 5:**
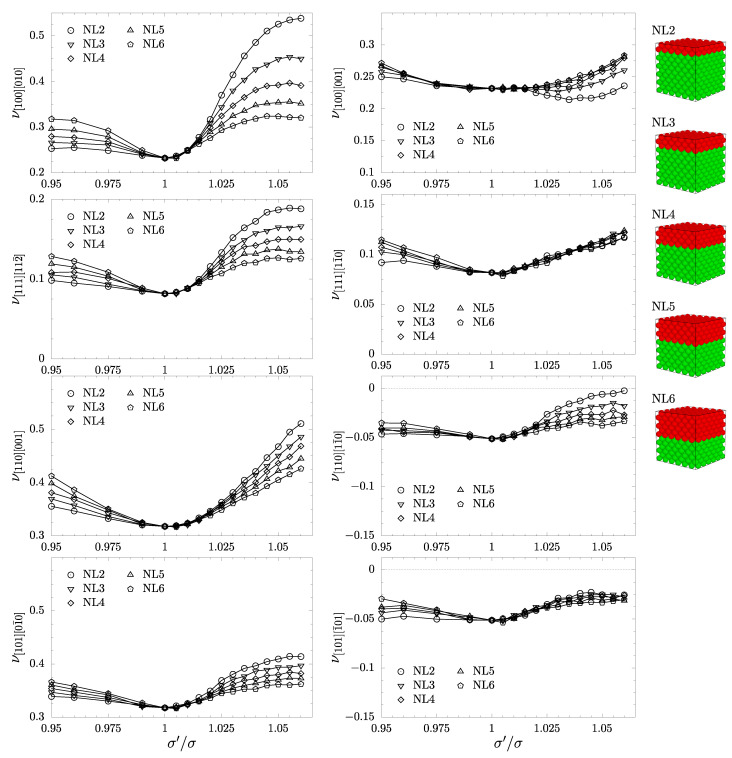
Poisson’s ratio in the main crystallographic directions as a function of $\sigma^{\prime }/\sigma$ for neighboring nanolayer systems.

**Figure 6:**
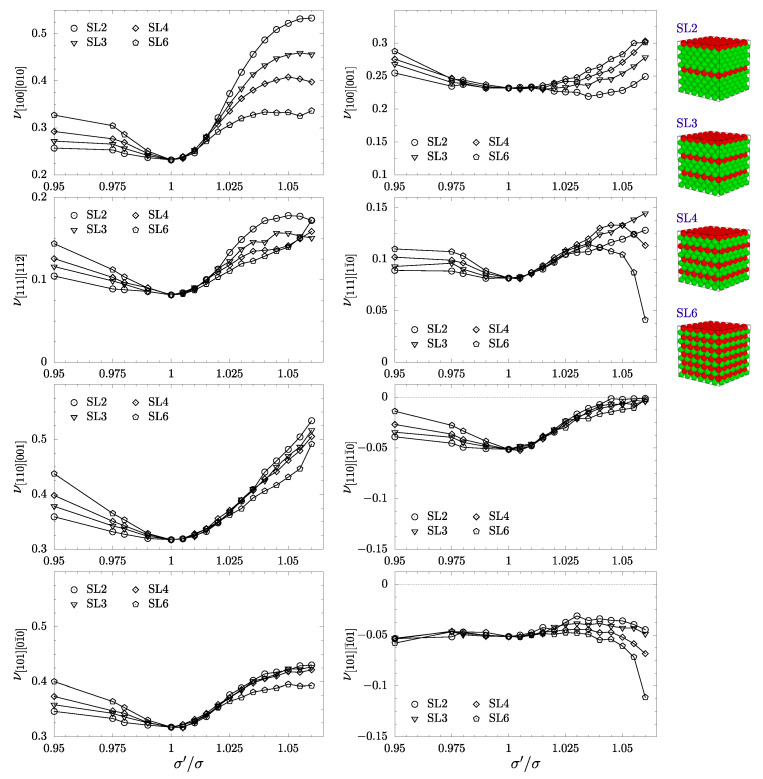
Poisson’s ratio in the main crystallographic directions as a function of $\sigma^{\prime }/\sigma$ for separated nanolayer systems.

To investigate how the layer inclusions’ ordering and their numbers impact Poisson’s ratio for the studied systems, we plot Poisson’s ratio in the main crystallographic directions as a function of $\sigma^{\prime }/\sigma$ in Figure 5 for neighboring layer systems and in Figure 6 for separated layer systems. Since all the systems when $\sigma^{\prime }/\sigma \neq 1$ have no cubic symmetry, the elastic properties in directions [100] and [111] are no longer isotropic. Therefore, Poisson’s ratio measured in [010] and [001], when the load is applied in the [100] direction, will differ. It can be seen in Figure 5 that added inclusion layers cause a significant increase in $\nu_{\left[100][010\right]}$ and $\nu_{\left[111\right]\left[11\bar{2}\right]}$ with $\sigma^{\prime }/\sigma$. The magnitude of the increase is inversely proportional to the number of layers added. The PR value of the system with the smallest, two-layer inclusion at $\sigma^{\prime }/\sigma =1.06$ is almost double in comparison to the PR of a pure hard sphere crystal. In the directions orthogonal to the former two, respectively, ($\nu_{\left[100][001\right]}$ and $\nu_{\left[111\right]\left[1\bar{1}0\right]}$), we observe the general tendency for the PR value to increase and see rather weak changes of the PR value with the number of layers added. Only in the case of the NL2 system is a brief decrease in PR in the range of $1.02\leq \sigma^{\prime }/\sigma \leq 1.055$ for the [100][001] direction observed. When looking at the PR in the [110] and [101] directions, which are equivalent in a system with cubic symmetry, one can see a similar behavior to $\nu_{\left[100\right]}$ and $\nu_{\left[111\right]}$. For all cases of $\sigma^{\prime }/\sigma \neq 1$, Poisson’s ratio in both directions is higher than in a system with cubic symmetry. In the $\left[101\right]\left[\bar{1}01\right]$ direction, one can note the weak dependence of PR on the number of inclusion layers added, in contrast to directions [110][001], $\left[110\right]\left[1\bar{1}0\right]$, and $\left[101\right]\left[0\bar{1}0\right]$. In the case of the NL2 system, the partial auxeticity in the $\left[110\right]\left[1\bar{1}0\right]$ direction is almost removed at $\sigma^{\prime }/\sigma =1.06$. Interestingly, the introduction of nanolayers into the system has a weak influence on the auxetic properties in the $\left[101\right]\left[\bar{1}01\right]$ direction.

In the PR plots concerning separated layer systems (Figure 6), one can observe that data for the two-layer system (SL2) is almost identical to the NL2 system. The only difference can be seen for $\nu_{\left[101\right]\left[\bar{1}01\right]}$, where the values of PR at higher $\sigma^{\prime }/\sigma$ are lower than for the NL2 system. Poisson’s ratio of the remaining SL*x* systems also closely follows their NL*x* counterparts, with only a few differences. On the $\nu_{\left[110\right]\left[1\bar{1}0\right]}$ plot, one can no longer see a significant correlation between the number of inclusion layers and the growth of the PR value. The auxetic properties in this direction almost disappear for $\sigma^{\prime }/\sigma$ bigger than approximately 1.04. A more interesting behavior of PR can be seen in the plots of $\nu_{\left[111\right]\left[1\bar{1}0\right]}$ and $\nu_{\left[101\right]\left[\bar{1}01\right]}$, where PR decreases for SL4 (four-) and SL6 (six-layer) systems compared to their respective NL*x* counterparts. One can see that auxetic properties for the SL6 system are notably enhanced compared to NL6 and to pristine cubic HS crystal.

**Figure 7:**
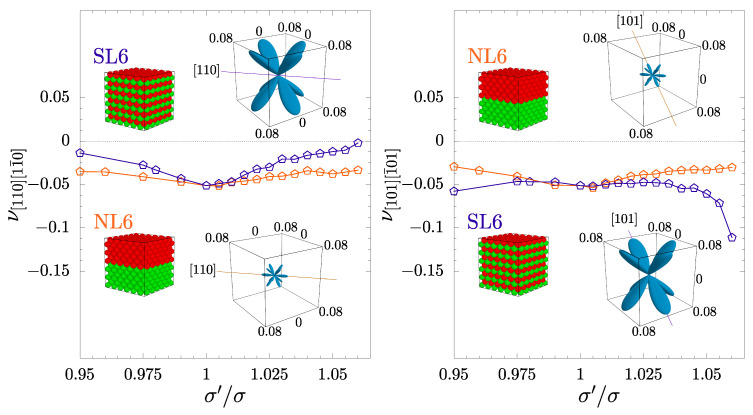
The comparison of Poisson’s ratio in auxetic directions $\left[110\right]\left[1\bar{1}0\right]$ and $\left[101\right]\left[\bar{1}01\right]$ (the results corresponding to $\left[011\right]\left[0\bar{1}1\right]$ are the same as for $\left[101\right]\left[\bar{1}01\right]$) for six nanolayer systems (NL6 vs. SL6). The inserts in the figures present the absolute value of minimal negative Poisson’s ratio in all crystallographic directions plotted in spherical coordinates for both systems at $\sigma^{\prime }/\sigma =1.06$. The solid line in the inserts shows the considered crystallographic direction.

In Figure 7, the comparison of Poisson’s ratio of systems with six separated (SL) and neighboring layers (NL) is made. Given the same concentration of inclusion particles (c=50%), one can observe how the change in spatial ordering of the inclusion layers impacts the elastic properties of the system. Poisson’s ratio in two auxetic directions, $\left[110\right]\left[1\bar{1}0\right]$ and $\left[101\right]\left[\bar{1}01\right]$, is presented. Additionally, the absolute value of the negative part of the minimal Poisson’s ratio calculated in all crystallographic directions for both SL and NL systems plotted in spherical coordinates has been included as graphical inserts. In the left part of Figure 7, one can see that the PR of the SL6 system in the $\left[110\right]\left[1\bar{1}0\right]$ direction grows up with $\sigma^{\prime }/\sigma$ and almost approaches zero for $\sigma^{\prime }/\sigma =1.06$, whereas the value of PR of the NL6 system changes slightly and maintains a negative value for all considered $\sigma^{\prime }/\sigma$. On the other hand, the PR of the SL6 system in the $\left[101\right]\left[\bar{1}01\right]$ direction significantly decreases its value (see the right part of Figure 7), while the value of PR in the $\left[101\right]\left[\bar{1}01\right]$ direction of the NL6 system remains almost unchanged and close to values of PR in the $\left[110\right]\left[1\bar{1}0\right]$ direction. This effect is also reflected in the graphical inserts with the minimal PR plots. Here, one can observe the enhancement of the auxetic properties due to the layer inclusion ordering in hard sphere crystals.

It is also worth noting that the value of the auxeticity parameter [78] for the SL6 system equals to $1.6\times 10^{-2}$, which, compared with the auxeticity parameter for the NL6 system ($2.4\times 10^{-3}$), is around 7 times higher. It clearly shows the possibility of essential changes in the auxetic properties of the system due to the ordering of layer inclusions in crystals.

Although the study presented here is purely theoretical, it provides qualitative results showing possible material behaviors. Fundamental research is the starting point for experiments on real systems and for predicting physical phenomena. We consider systems where particles interact with the simplest possible interaction potential used in condensed matter theory. Hard potential is non-analytical, yet research using it provides us with the opportunity to observe new interesting effects, including those discussed in this paper. The behavior of models presented in this work can occur in real systems, for example, at high pressures when interactions between atomic cores prevail.

## 5. Conclusions

The elastic properties of the hard sphere crystal with multiple nanolayer inclusions formed by spheres of another diameter were determined using Monte Carlo simulations in the NpT ensemble. The layer inclusions were introduced in a selected crystallographic plane orthogonal to the [001] direction. Systems with neighboring inclusions and systems with separated inclusions were considered.

The introduction of inclusions in the form of nanolayers into the pure hard sphere crystal strongly impacts its elastic properties. The studies showed that both the size of inclusion spheres and the number of layers and their spatial ordering are critical factors in the modification of elastic properties of the hard sphere crystal. It was found that increasing the number of layers in the system leads to more significant changes in the elastic properties of systems with neighboring layer inclusions than separated ones.

An important observation concerns the auxetic properties of the studied systems. It was found that, by merely changing the spatial ordering of the inclusions in the six-layer system such that the individual layers are separated from each other, one can achieve strong enhancement of auxetic properties in the $\left[101\right]\left[\bar{1}01\right]$ direction. Moreover, Poisson’s ratio in this direction is lower than in the system with neighboring layers, as well as in the pristine hard sphere crystal. However, the auxetic properties of the six separated layers system in the [110] direction almost disappear. Another interesting observation is that Poisson’s ratio of the six neighboring layers system in the auxetic directions is negative and almost unchanged. This system maintains its auxetic properties, independent of the size of the spheres forming the inclusions.

The most important finding of this study is that the spatial ordering of nanolayer inclusions formed by spheres of another diameter allows one to tune the auxetic properties of the system. Finally, it is worth adding that modern nanotechnology already allows for obtaining monatomic layers [72], thus the ideas and results presented in this work may inspire other researchers working in nanoscience to implement layered structures in practice to achieve metamaterials with desired auxetic properties. These unique properties may be used in some applications of auxetic metamaterials that were recently reviewed [79]. In closing the paper, it is worth pointing out that, without simulations, it is very difficult (if at all possible) to predict how a specific modification of the structure will affect the elastic properties of the crystal. Currently, studies of a number of different models with different combinations of inclusions are being conducted, and the results of these studies will be presented in future publications.

## Figures and Tables

**Table 1:** The parameters for the studied models. The graphical inserts schematically illustrate the particular layer inclusion ordering within the unit supercell. The concentration values are calculated for the system size of 6×6×6 FCC unit cells.

Model Name	Layer Ordering (dir. [001]→)	Inclusion Layers	$N_{\mathrm{inc}}$	c[%]
NL2	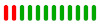	2	144	16.23
NL3	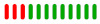	3	216	25.23
NL4	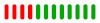	4	288	33.13
NL5	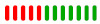	5	360	41.23
NL6	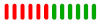	6	432	50.23
SL2	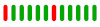	2	144	16.23
SL3	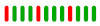	3	216	25.23
SL4	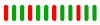	4	288	33.13
SL6	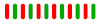	6	432	50.23

## Data Availability

Data is contained within the article.

## Abbreviations

The following abbreviations are used in this manuscript (listed in order of occurrence in the text):

PR	Poisson’s ratio
HS	Hard Sphere
FCC	Face-Centered Cubic
NL	Neighboring Layers
SL	Separated Layers
MC	Monte Carlo
